# Malignant schwannoma of the infratemporal fossa: a case report

**DOI:** 10.1186/s13256-015-0624-6

**Published:** 2015-07-04

**Authors:** Mohamed Mliha Touati, Youssef Darouassi, Mehdi Chihani, Abdelfettah Al Jalil, Khalid Tourabi, Mohamed Lakouichmi, Ismail Essadi, Brahim Bouaity, Haddou Ammar

**Affiliations:** 1 ENT Department, Military Hospital Avicenna, Avenue Al Mouqaouama, Marrakech, 40000 Morocco; 2 Stomatology and Maxillofacial Surgery Department, Military Hospital Avicenne, Avenue Al Mouqaouama, Marrakech, 40000 Morocco; 3 Medical Oncology Department, Military Hospital Avicenne, Avenue Al Mouqaouama, Marrakech, 40000 Morocco

**Keywords:** Malignant schwannoma, Infratemporal fossa, Paranasal sinuses, Immunohistochemistry

## Abstract

**Introduction:**

Malignant schwannomas or neurofibrosarcomas are rare nerve tumors of unknown etiology. These neoplasms are highly aggressive with a marked propensity for local recurrence and metastatic spread. Their management continues to be a challenge for pathologists and surgeons. Maxillofacial locations are very exceptional. We report the case of a patient with unusual malignant schwannoma of the infratemporal fossa discovered at a late evolving stage.

**Case presentation:**

A 56-year-old woman, of Moroccan nationality, presented to our hospital in 2013 with a large right-sided hemifacial swelling that had evolved over the previous 4 months, with a limitation of mouth opening, nasal obstruction and episodes of epistaxis. A CT scan and MRI showed a large and invasive tumor occupying her right infratemporal fossa and maxillary sinus, with sphenoidal, ethmoidonasal, nasopharyngeal and intraorbital extension. A nasal endoscopic biopsy was performed. Immunohistochemical examination concluded a diagnosis of malignant schwannoma, and a palliative radiotherapy was decided; however, our patient died 10 days later.

**Conclusions:**

Malignant schwannoma of paranasal sinuses and the anterior skull base is a rare tumor that involves a high rate of local invasion. The prognosis is poorer compared to that occurring in the trunk and extremities.

## Introduction

Malignant schwannomas or neurofibrosarcomas are rare nerve tumors with very poor prognosis; maxillofacial locations are very exceptional [1]. These tumors may be either primitive or as a complicating part of neurofibromatosis type 1 (NF-1), also known as von Recklinghausen’s neurofibromatosis [1]. We report an exceptional case of malignant schwannoma of the infratemporal fossa discovered at a late evolving stage, with a large extension to the neighboring structures, and discuss, through a literature review, the various features of this tumor.

## Case presentation

A 56-year-old woman, of Moroccan nationality, a chronic cigarette smoker consuming an average of one pack a day for 38 years, was hospitalized for a large right-sided hemifacial swelling. Her early symptoms dated from 4 months previously, by the appearance of straight facial neuralgia, treated as dental neuralgia without any improvement. The evolution was marked by rapid aggravation of the pain, which then became disabling, and the emergence of a right-sided maxillary swelling rapidly increasing in size and accompanied by a limitation of mouth opening, and a nasal obstruction with episodes of epistaxis.

A clinical examination determined a painful and inflammatory right-sided hemifacial swelling, right exophthalmus without notion of decreased visual acuity, and hypoesthesia at the second and third trigeminal nerve areas. An endonasal examination detected a tumor mass in her right nasal cavity, friable and bleeding at contact. An oral examination, very difficult because of the limited mouth opening, showed an erosion of the hard palate. Cervical lymph node areas were free and an endoscopy examination of the upper aerodigestive tract did not show any lesions. Otoscopy and audiometry test results were normal. Our patient was hospitalized and placed immediately on morphine.

A computed tomography (CT) scan revealed the presence of a large heterogeneous mass occupying her infratemporal fossa as well as the masticator space and right maxillary sinus, lysing the walls of the maxillary sinus, the ascending branch of the mandible and the orbital floor with ethmoidonasal, parapharyngeal and intraorbital extension.

**Fig. 1:**
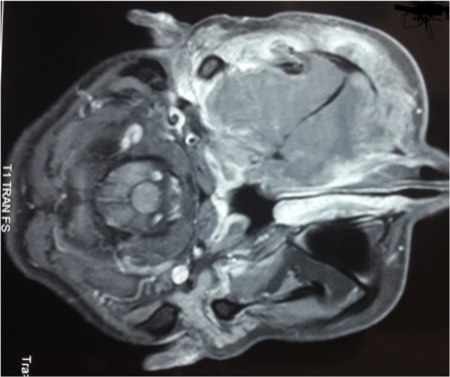
Axial T1-weighted magnetic resonance image of the facial area showing an invasive tumor occupying the right infratemporal fossa and maxillary sinus with irregular boundaries, heterogeneous intermediary signal at T1 weight, with sphenoidal, ethmoidonasal, and nasopharyngeal extension

**Fig. 2:**
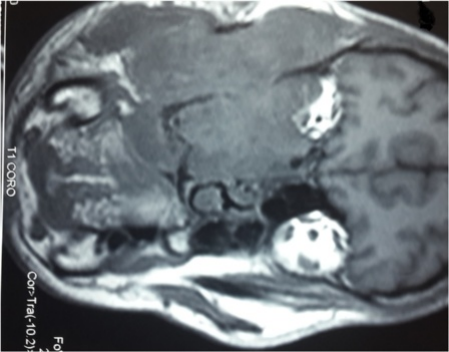
Coronal T1-weighted magnetic resonance image of the facial area showing an invasive tumor occupying her right infratemporal fossa and maxillary sinus with irregular boundaries, heterogeneous intermediary signal at T1 weight, with sphenoidal, ethmoidonasal, nasopharyngeal extension

**Fig. 3:**
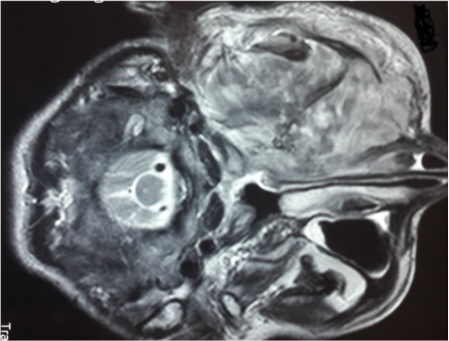
Axial T2-weighted magnetic resonance image of the facial area showing the ethmoidonasal, and intraorbital extension, with infiltration of the jugal and temporozygomatic soft parts

**Fig. 4:**
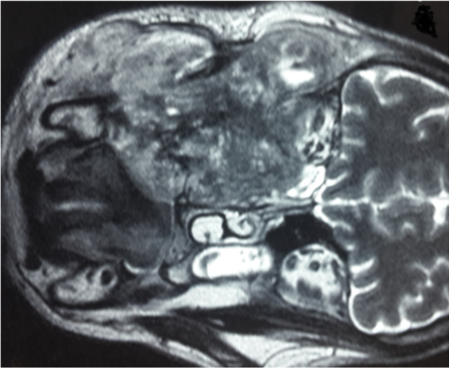
Coronal T2-weighted magnetic resonance image of the facial area showing the ethmoidonasal, and intraorbital extension, with infiltration of the jugal and temporozygomatic soft parts

**Fig. 5:**
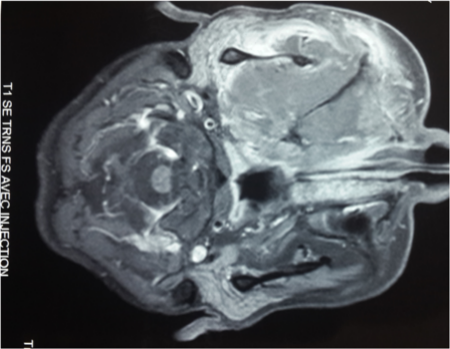
Axial T1-weighted magnetic resonance image of the facial area showing heterogeneous contrast enhancement and necrotic areas

**Fig. 6:**
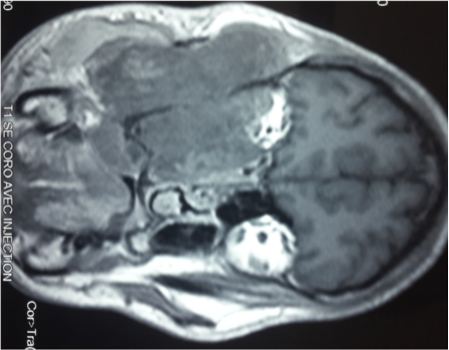
Coronal T1-weighted magnetic resonance image of the facial area showing heterogeneous contrast enhancement and necrotic areas

A magnetic resonance imaging (MRI) scan showed an aggressive and invasive tumor occupying her right infratemporal fossa and maxillary sinus with irregular boundaries, heterogeneous intermediary signal at T1 and T2 weight, with sphenoidal, ethmoidonasal, nasopharyngeal and intraorbital extension. This process infiltrated the jugal and temporozygomatic soft parts, with heterogeneous contrast enhancement and areas of necrosis (Figs. 1, 2, 3, 4, 5 and 6).

**Fig. 7:**
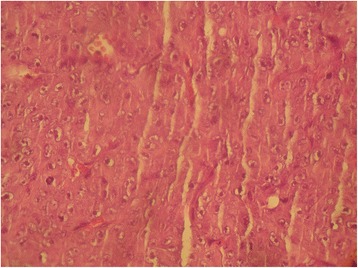
Malignant tumoral proliferation, poorly differentiated and infiltrating. (H&E × 100)

**Fig. 8:**
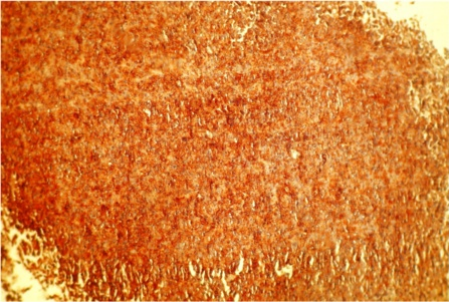
Positive and diffuse cytoplasmic immunostaining of tumor cells anti-S100 protein antibody, confirming the diagnosis of malignant schwannoma. (H&E × 200)

A nasal endoscopic biopsy was performed. A pathological examination was in favor of a malignant process, poorly differentiated and invasive (Fig. 7). An immunohistochemical examination demonstrated a total cytoplasmic positivity to anti-S100 protein antibody, concluding in a diagnosis of malignant schwannoma (Fig. 8). The staging, including a cervico-thoraco-abdominal CT scan, did not find any distant metastasis. According to the clinical and radiological data obtained, palliative radiotherapy was decided, delivered by a linear electron accelerator, at a total dose of 30Gy, 10Gy by fraction, over 3 days, with one field including the tumor mass. The outcome was favorable with improvement of symptoms. Unfortunately, our patient died 10 days later after severe respiratory distress.

## Discussion

Malignant schwannoma, also called neurofibrosarcoma, neurogenic sarcoma or malignant tumor of the peripheral nerve sheath (malignant peripheral nerve sheath tumors: MPNSTs), is a tumor of the peripheral nervous system that develops in the nerve sheath at the expense of Schwann cells [1]. This tumor is rare with an estimated incidence of 0.1 per 100,000 per year in the general population [1, 2].

It accounts for approximately 5 to 10 % of all soft tissue sarcomas and has a strong association with NF-1 [2, 3], also known as von Recklinghausen’s neurofibromatosis. Up to 30 to 50 % of all MPNSTs are found in association with NF-1 [2]. Radiation has been also implicated as an etiologic factor in the development of MPNST with a latency interval of 10 to 20 years [4]. Malignant degeneration of benign schwannoma seems to be a rare situation and even exceptional [2, 3].

MPNSTs represent only 2 to 6 % of head and neck sarcomas [4], evolution of the MPNST into paranasal sinuses or skull base is extremely rare [3, 5].

The origin of most MPNSTs of the nose, the paranasal sinuses and the anterior skull base is presumed to be the ophthalmic and maxillary divisions of the trigeminal nerve and its terminal branches, as well as autonomic ganglia [3, 5].

These tumors primarily affect adults aged between 20 and 50, with an average of 40 years of age, without sex predominance [2]. The forms associated with NF-1 occur late and affect mainly men [2, 3].

Clinically, MPNSTs generally are presented as a progressively enlarging and painful mass in the paranasal sinus or anterior skull base. Malignant schwannomas may become clinically apparent with unilateral nasal obstruction, hyposmia, epistaxis, atypical pain, hypoesthesia or localized swelling of the facial and orbital region, mucopurulent rhinorrhoea and headache [6].

Exophthalmus, epiphora and progressive visus reduction are less frequently described and are principally related to neoplasms of the orbital compartment. Intracranial extension has also been reported [5, 6].

High-resolution CT scanning is considered to be an adequate imaging investigation for schwannomas of paranasal sinuses [7]. CT, however, is crucial to a preoperative surgical plan, revealing the pushing borders of the tumors, rather than the invasive character of malignancy [7, 8]. There is generally no bone development; erosion of the neighboring bone structures may rarely be observed secondarily to necrosis caused by the pressure of the growing mass [7]. MRI with gadolinium contrast may provide more useful information, and is indicated in areas with intraorbital or intracranial extension, and for more exact delineation of the tumor from normal soft tissue. Even in situations where the schwannoma is confined within the sinuses and nasal cavity, MRI is helpful in differentiating the neoplasm from retained secretions or inflammatory changes [8].

Microscopically, MPNSTs are highly cellular, focally polymorphic tumors compounded of spindle cells arranged in bundles or fascicles with high mitotic rates, indistinct cytoplasmic borders and a variable degree of nuclear pleomorphism [5, 9].

MPNSTs can be histologically similar to other malignant tumors, particularly malignant melanoma and other spindle cell sarcomas. Currently, immunohistochemical analysis especially, but also the recognition of Schwann cells by electron microscopy, help identify MPNSTs [9, 10]. In immunohistochemical analysis, the majority of MPNSTs express the neuroectodermal marker S-100 protein and the mesenchymal marker vimentin, while cytokeratin and desmin are rarely identified [9, 10].

A literature review by Pfeiffer, revealed that of 33 well-documented cases of MPNSTs localized in the anterior skull base or the paranasal sinuses published since 1971 [10], only nine cases had MPNSTs in both locations (as our patient).

Management of MPNSTs has often been reported to be challenging, especially in the head and neck, so early diagnosis is indispensable. The recommended treatment of MPNSTs is surgical extirpation including wide margins, and involved nerves should be followed proximally in an attempt to obtain clear margins. Regional nodal dissection is not recommended. The surgical approach depends upon the location and the extent of the tumor [11].

The role of radiotherapy and chemotherapy in the treatment of this tumor is still controversial. MPNST has traditionally been described as being highly radioresistant, but, some recent reports recommend the use of postoperative radiotherapy, but there is no clear evidence of definite advantage [10, 11].

For our patient, the location of the tumor in the infratemporal fossa and the large infiltration of neighboring structures made it difficult to perform surgery, and treatment was limited to radiotherapy.

MPNST is a very aggressive tumor that spreads via direct perineural invasion and the hematogenous route. The local recurrence rate is about 54 % and the rate of distant metastases to lung and bone is about 65 %. Lymph node involvement is not common [6, 11].

The prognosis of MPNSTs of the head and neck is relatively poorer than that of MPNSTs of the extremities and the trunk with documented 5-year survival rates from 15 to 35 % [2, 11], these tumors have even closer proximity to vital structures making local control by radical resection more critical. Due to the anatomical location, en bloc resection is often impossible and fractional excision is common.

## Conclusions

MPNST of the paranasal sinuses and the anterior skull base is a rare tumor that involves a high rate of local invasion, distant spread, and local recurrence.

The management of this aggressive neoplasm in this anatomical location remains an interdisciplinary challenge. A good outcome is mainly a function of local control by means of surgical resection. The prognosis is poorer compared to that occurring in the trunk and extremities.

## Consent

Written informed consent was obtained from the patient’s next-of-kin for publication of this case report and any accompanying images. A copy of the written consent is available for review by the Editor-in-Chief of this journal.

## Abbreviations

CT: computed tomography
MPNSTs: malignant peripheral nerve sheath tumors
MRI: magnetic resonance imaging
NF-1: neurofibromatosis type 1
